# Perspectives from the periphery of Paediatrics

**DOI:** 10.3389/fpsyg.2022.1044692

**Published:** 2022-12-06

**Authors:** Zainab Ahmed Alani

**Affiliations:** University of Glasgow Medical School, University of Glasgow, Glasgow, United Kingdom

**Keywords:** rare disease (RD), generalised Myasthenia Gravis, paediatrics, Myasthenia Gravis, patient – centred care, paediatric to adult services, paediatric to adult care services, equality

## Introduction

The gap between paediatric and adult care is, undeniably, colossal. Without a doubt, navigating the journey between these vastly different domains of care can prove extremely challenging for all involved; whether this be patients, parents and carers, or clinicians themselves. However, having personally traversed this avenue filled with uncertainty as a patient battling the rare condition generalised Myasthenia Gravis, I believe the toll of this task to be doubly wounding for those with rare diseases. Not only is the prospect of a daunting diagnosis now looming, but expectations to acclimatise to new healthcare environments heighten tensions to such an extent that the mental stress and adaptiveness expected of patients is now unfathomable. Nevertheless, the lack of continuity in care between paediatric and adult care is disconcerting for all patients, regardless of diagnosis. This article explores for whom and, vitally, how healthcare systems have failed to provide adequate levels of care due to pitfalls in paediatric care frameworks. Additionally, having witnessed first-hand several of the hurdles hindering this transition, and with my valuable insider-outsider perspective as both a generalised Myasthenia Gravis patient and medical student, I believe there exists efficacious, yet achievable, methods capable of eliminating such surmountable drawbacks. Just as maturing paediatric patients are expected to adjust to new environments, approaches and healthcare providers, we too must collectively modify healthcare frameworks in a perceptive manner to better support patients making this seismic transition. Undoubtedly, such approaches aiming to better integrate paediatric and adult care will promote a more inclusive care environment which in turn fosters and promotes greater patient engagement. Moreover, for patients battling rare disease diagnoses, this approach will unequivocally dampen some of the overwhelming anxiety and uncertainty that may linger. Only by working in unison, and utilising both a child's ingenuity and an adult's intuitiveness, can we pave the way for a smoother and more secure journey for patients progressing from paediatric to adult care.

Age is a curious thing. Worldwide, this objective measurement is a universal language accepted by all without quarrel or controversy. Compared to other personal characteristics including race, religion, and gender, we place particular importance on age in our modern world and place it on a pedestal. The reasoning for this may lie in the fact that, unlike its counterparts, age is an undeniable constant; it cannot be changed and proceeds on its unwavering path regardless of any and all human intervention. We use it to decide at which age we should be allowed to accept responsibility. We use it to establish education systems; subdividing each stage with corresponding expected standards. Vitally, however, age is used globally to delineate our healthcare systems into paediatric and adult care, in acknowledgement of the differences between child and adult health. Yet, despite age playing such a crucial role in distributing healthcare, it appears that there is a worldwide struggle in truly defining the term ‘paediatrics' (Kahn et al., 2014; Clark et al., 2015; Hirschfeld, 2015). When considering the epistemology of paediatrics, we must acknowledge the limitations of human categorisation of paediatrics as problems may arise such as when those categorized as “paediatric patients” may be deemed able to give consent before they are classified as adults (Williams and Perkins, 2011). According to the NHS, 2022, paediatric care is defined as “*covering children from birth to the age of 16”* clearly outlining, at least on paper, the boundaries between child and adult health. However, how well does this written definition translate to real-life clinical practise when patients are neither young children nor adults, and find themselves straddling between these subdivisions in the healthcare system's no man's land?

Stereotypically, adolescents are seen as difficult with clinicians harbouring preformed conceptions (Hirschfeld, 2015); however this may be due to lack of sufficient training, understanding, and experience with such patients rather than implicit or explicit bias (Bennett et al., 2009; AlBuhairan, 2014; Manassis, 2014; Takeuchi et al., 2021). They do not do what they are told; they are emotional because of hormonal changes; they follow their own rules, coupled with an intrinsic propensity to rebel. Despite these grossly misdirected assumptions, it is indeed true that teenagers and young adults – already undergoing a time of change in their lives – often have very different needs and, as such, provision of healthcare must be approached differently (Nursing Times, 2014). Simultaneously, this does not mean that healthcare standards should be any different and thus, the quality of care for adolescents must equate to the admirable standards set out for paediatric care as a whole (Royal College of Paediatrics and Child Health, 2018). Looking at the bigger picture, adolescents are still encompassed within the umbrella term of paediatrics. Surely then, they deserve the same high-quality treatment as their younger counterparts? Unfortunately, this is not the case, with young adults having worse outcomes than other users of both paediatric and adult care (Albon and Vaughan, 2014). Those aged between 15 and 19 have greater mortality rates than those aged 1–4 and those aged 20–24 (Albon, 2016). Furthermore, a study in the BMJ found that over a third of young adults lost their successful kidney transplant when transitioning from paediatric to adult care (Harden et al., 2012). Additionally, a 2012 study found that patients aged between 17 and 24 had greater median hospital stays compared to those aged 16 and below at 2 days and 1 day, respectively (Heaton et al., 2013). Although small, this gap should be non-existent as every extra second, minute and hour spent in hospital environments place additional anxiety on patients, parents and carers (Australian Psychological Society, 2016). Such powerful psychological impacts can mentally scar patients, leaving a painful reminder that lingers long after any physical wounds may heal. Contrary to popular belief, young people do not enjoy time away from school but rather, it has been shown that a return to normalcy and resumption of routine is beneficial to both patients and carers (Commodari, 2010). These aforementioned figures are deeply concerning and, today, such preventable failures should not exist. Clearly, it cannot be denied that there is a colossal gap in current frameworks which fail to facilitate a smooth and, more importantly, safe transition for young people.

Facts and figures speak volumes and unequivocally emphasise that there is a serious and unignorable issue deeply rooted within our healthcare system. Yet, as a patient living with the rare condition generalised Myasthenia Gravis (MG) who has had to endure this failed transition first-hand, I know that nothing communicates the extent and impact of an issue like lived experience. Although many view the worlds of patients and healthcare professionals as completely disconnected entities, and that those with dual representation may interpret studies differently (Dwyer and Buckle, 2009), I believe that the dichotomy is not that great and that experience in both environments has afforded me a unique view regarding this matter. When I presented to the paediatric A&E unit with a myriad of puzzling symptoms, I was 15 years old. However, I was nearer 16 and on the verge of adulthood and so too, on the verge of adult care. As such, my family and I were torn as to which service I should present to for seeking help, again highlighting the gross lack of guidance at such vulnerable ages for young people. The fear of the unknown and the urgency of these frightening symptoms took over, and I sought refuge in the comfort of the paediatric A&E unit where I had gone countless times before during my adventurous childhood. Whether I had broken my wrist on monkey bars or gotten a pencil lead in my foot, the bubbly environment and colourful walls magically seemed to plaster a smile on my face every time without fail. Yet this time, it wasn't the same. I received questioning looks, from a multitude of healthcare professionals, so powerful that their eyes communicated the message as if to say, “*You're no longer a child; why are you here?*” I had now grown too tall to fit the small chairs. The box of toys no longer beckoned. The building that once seemed so full of life and bursting with laughter had lost its charm; a vacuum had sucked away all life leaving despair in its wake, and I too was being drawn in. This is the periphery of paediatrics.

After countless visits, tests, and scans, no one knew what was wrong with me. As days and weeks passed, I was sent from pillar to post and pushed further and further to the fringes of paediatric care. Like an unwanted burden, I was sent between paediatric and adult services to no avail; after all, who wants to be responsible for the care of a patient with no discernible diagnosis? After an unenviable diagnosis journey, when I received my life-changing diagnosis of MG at the age of 15 years and 348 days, my journey of uncertainty and sense of no belonging abruptly ended as I was sent to adult care services; there was no smooth transition, no apparent framework, and no meaningful support. Suddenly, the caricatured walls of paediatrics were replaced with clinical white; the colourful plastic chairs were replaced with sombre fabric upholstered seats; the toy box was replaced with leaflets. Needless to say, this was doubly wounding; not only was this burden of a lifelong diagnosis robbing me of my childhood and forcing me into maturity, but the healthcare system failed to bridge this gap and inadvertently plunged me head-first into the deep end of adult care. With the whirlwind of events and an unshifting incurable diagnosis now looming, a frightening, unfamiliar environment was the dramatic finale of this rare disease rollercoaster.

Sadly, my experience of being met with such an extreme, sudden shift in the care environment is mirrored by many (Department of Health, 2003). Moreover, despite patient experiences from over half a decade ago signposting these inherent issues (Care Opinion, 2016), it appears that no impactful change has been implemented. A loss of sense of belonging is potent which, undeniably, has adverse effects on mental wellbeing; this is amplified for adolescents as they are already undergoing a time of drastic, unparalleled change (Fegran et al., 2014). At the same time, this proves to be not only an emotional struggle but also physically detrimental with adverse outcomes manifesting as a result of such uncoordinated transitions (Willis, 2020). A study investigating this issue regarding adolescents with Type 1 Diabetes found that patients were greatly disillusioned with discrepancies in follow-up experiences, with particular disappointment in adult care (Iversen et al., 2019). Furthermore, young people cared for in paediatric wards had greater satisfaction rates than those in adult wards at 54% and 44%, respectively, outlining once again the lack of communication and support regarding changes in care (Heaton et al., 2013). It mustn't be overlooked that, with this shift, a huge responsibility is now placed on the young person; adults, in most cases, must make their own healthcare decisions and a sudden shift to this environment with such expectations can initially be daunting. Despite recommendations for overlap of paediatric and adult services during this vulnerable time and implementation of audits to support young people make this transition, there is no clear strategy utilising the power of patient voice (Brooks et al., 2017). Undoubtedly, this is the key which will unlock the door to a smoother transition journey as it is only with patient views and expectations can we provide the necessary care experience.

**Figure 1:**
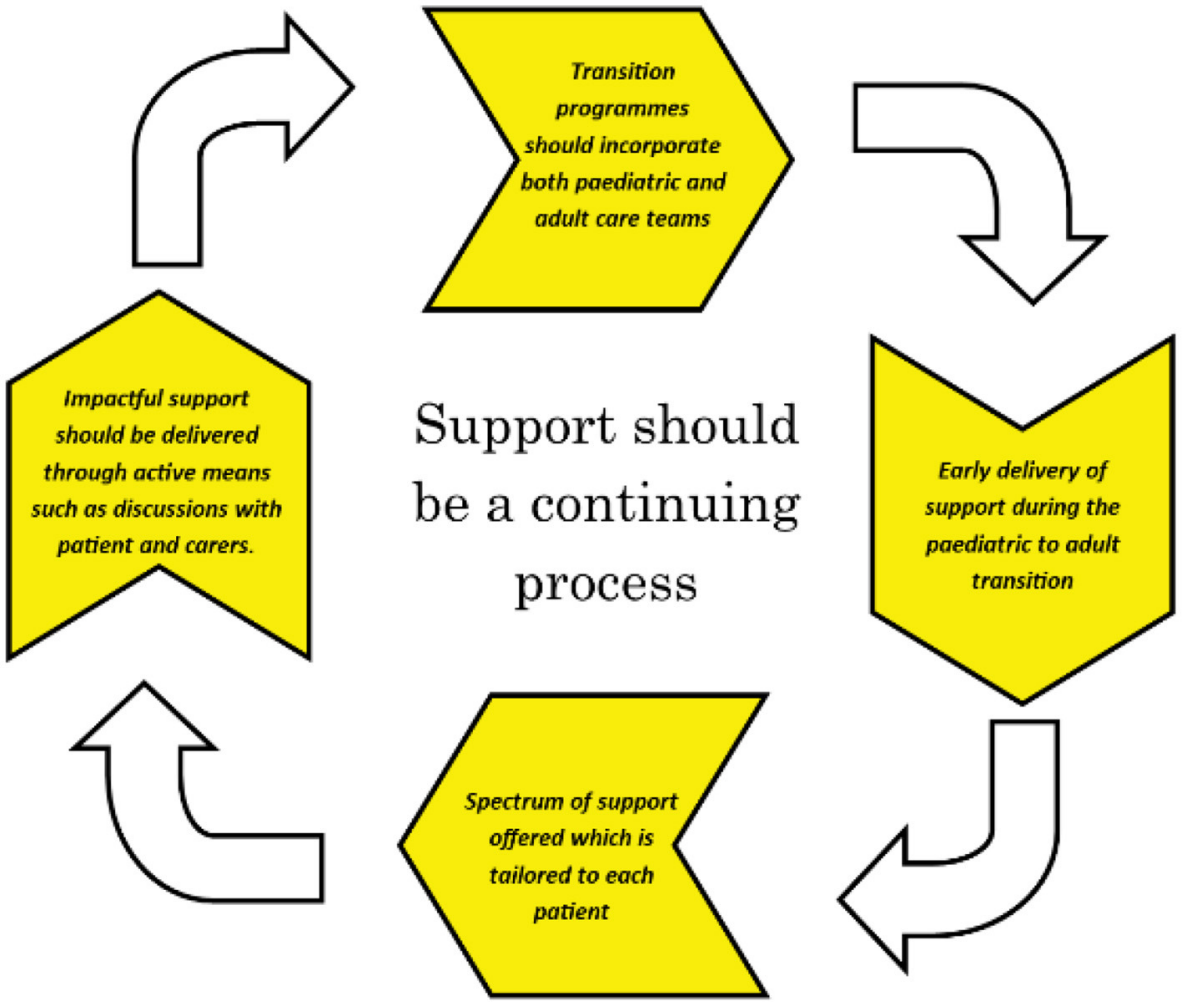
Support should be a continuing process ensuring early delivery of a spectrum of impactful support which is tailored to each individual patient's unique needs; implementation of transition programmes should incorporate both adult and paediatric care teams (created by Alani ZA, 2022).

In today's modern, technologically advanced world, networking and forming connextions have never been easier. With the advent of the coronavirus pandemic, there have been innumerable deaths and losses, but one positive that emerged was the accelerated and enhanced incorporation of telemedicine into medical practise (Vidal-Alaball et al., 2020). Even in the field of paediatrics, where there is powerful emphasis on thorough procedures to maximise detection of preventable illnesses presenting in childhood, telemedicine was seamlessly incorporated into clinical practise and offered advantages regarding diagnosis accuracy (Metzger et al., 2021). Therefore, having successfully established such measures worldwide to tackle this pandemic, we can employ similar strategies to combat the unspoken pandemic of treatment inequality within paediatric care. Such implementations could include measures as straightforward as online video calls between patients and carers and both paediatric and adult care providers, before making the transition. Thus, a connextion will be established early in the transition process so that patients feel they have adequate grounding from which to rebuild their new care expectations in the unfamiliar adult care environment. To ensure delivery of impactful support, transition programmes should not be once and for all but rather, should be a continuing process offering a spectrum of support from early on in paediatrics right through to when patients are in adult care (Figure 1) (Bryon and Madge, 2001). Additionally, interactive maps or videos of the patient's new environment could be provided before their first visit. In doing so, this simple approach removes one of the veils obscuring the world of adult care by taking the edge off initially stepping into unfamiliar environments. To harness the power of patient voice, young adult patients who have already moved to adult care could provide tailored advice to those currently going through the transition. As an MG patient advocate, I am aware of the intrinsic longing of patient advocates to help younger patients navigate their healthcare journey so that unnecessary distress can be avoided. With the growing positive influence of social media in raising awareness of health care issues including dissemination of information programs during the COVID-19 pandemic (Abuhashesh et al., 2021), finding and reaching out to patients willing to share their invaluable experiences is undoubtedly achievable. Moreover, signposting patients to such services could be easily facilitated as there are already such successful platforms from which to project these indispensable services. Such simple yet realistic targets should be set so that, before any legislations are passed or white papers published, the patients of today experiencing these struggles can sense their calls for reform being reflected in real, meaningful change.

Revolutionising this transition is certainly achievable with encouraging results from similar transition programmes implemented amongst groups of sickle cell patients demonstrating the achievement of lower hospitalisation rates and elimination of emergency handovers to adult care (Hoegy et al., 2020). Another intervention into a separate group of sickle cell patients, which identified this elevated risk during the paediatric to adult transition to be associated with elements which were both physically and psychologically manifested in nature, sought to intervene to improve patient outcomes. It was identified that, in order for such programmes to be successful, they must be an ongoing activity which collaborates with patients, parents and the whole multidisciplinary team (de Montalembert et al., 2014). Meanwhile, others appreciate the importance of the psychological aspect during this transition and highlight that actions that patients can take themselves, such as increased independence and reliance alongside assessing the patient's preparedness for this transition, are also highly valuable (Inusa et al., 2020). Studies amongst cystic fibrosis (CF) patients have also identified the importance of increased involvement of patients, as well as their families, which contributed towards enhanced self-reported outcomes (Okumura et al., 2014). Similarly, amongst inflammatory bowel disease patients, it was identified that making care more individualised to each patient and taking into consideration all of the factors affecting their care and perceptions of health were vital (Nardone et al., 2020). As a rare disease patient myself, I too believe this to be one of the most important factors necessary for successful transition programmes as each individual's needs are starkly different, even though clinical diagnoses may appear identical at first glance.

Bringing down this invisible yet seemingly insurmountable barrier between paediatric and adult care means that we must establish protocols to better link these two services which, rather than existing as two separate entities, should be united within a single, flowing care spectrum. However, changes to the system alone will be inadequate for proper unification and we - clinicians, patients, and parents - must provide personalised support and encouragement for young people making this seismic transition. Collectively, we must work towards eliminating the striking differences in care and outcomes between young children and adolescents in paediatric care and ensure that all paediatric patients - regardless of age - receive the same high quality care. If we do not act now, the sequelae of such chronic negligence may prove irreversible. As a rare disease patient who has found themselves overwhelmed by these vastly contrasting environments, approaches, and layouts, I understand how vital such implementations would be not only for patients but also for parents witnessing their child endure such emotional distress. Achieving these goals would mean adolescents are no longer pushed to the periphery of paediatrics but rather, are embraced, welcomed, and eased in to the unnerving world of adult care. At a young age, the hospital environment itself is daunting enough, and needn't be compounded by a tortuous transition journey. Together, we can help young people navigate this no man's land and replace fear of enigmatic adult care with an insightful understanding of what the future holds.

## Author contributions

The author confirms being the sole contributor of this work and has approved it for publication.

## Funding

APC covered by Medics4RareDiseases.

## Acknowledgements

I would like to thank my family for their unfaltering support and continued encouragement, throughout both my personal and academic life, without whom I would be unable to fulfil my full potential.

## Conflict of interest

The author declares that the research was conducted in the absence of any commercial or financial relationships that could be construed as a potential conflict of interest.

## Publisher's note

All claims expressed in this article are solely those of the authors and do not necessarily represent those of their affiliated organizations, or those of the publisher, the editors and the reviewers. Any product that may be evaluated in this article, or claim that may be made by its manufacturer, is not guaranteed or endorsed by the publisher.
